# Regional Warming and Emerging Vector-Borne Zoonotic Dirofilariosis in the Russian Federation, Ukraine, and Other Post-Soviet States from 1981 to 2011 and Projection by 2030

**DOI:** 10.1155/2014/858936

**Published:** 2014-06-19

**Authors:** Vladimir Kartashev, Alexandr Afonin, Javier González-Miguel, Rosa Sepúlveda, Luis Simón, Rodrigo Morchón, Fernando Simón

**Affiliations:** ^1^Department of Infectious Diseases, Rostov State Medical University, Nakhichevanskiy Pereulok 29, Rostov-na-Donu 344022, Russia; ^2^Faculty of Geography, Saint Petersburg State University, Universitetskaya Nab. 7-9, Saint Petersburg 199034, Russia; ^3^Laboratory of Parasitology, Faculty of Pharmacy & IBSAL, University of Salamanca, C/del Licenciado Méndez Nieto s/n, 37007 Salamanca, Spain; ^4^Department of Statistics, University of Salamanca, C/Alfonso X el Sabio S/N, 37007 Salamanca, Spain

## Abstract

We analyze through a climatic model the influence of regional warming on the geographical spreading and potential risk of infection of human dirofilariosis in Russia, Ukraine, and other post-Soviet states from 1981 to 2011 and estimate the situation by 2030. The model correctly predicts the spatiotemporal location of 97.10% of 2154 clinical cases reported in the area during the studied period, identified by a retrospective review of the literature. There exists also a significant correlation between annual predicted *Dirofilaria* generations and calculated morbidity. The model states the progressive increase of 14.8% in the potential transmission area, up to latitude 64°N, and 14.7% in population exposure. By 2030 an increase of 18.5% in transmission area and 10.8% in population exposure is expected. These findings strongly suggest the influence of global warming in both geographical spreading and increase in the number of *Dirofilaria* generations. The results should alert about the epidemiological behavior of dirofilariosis and other mosquito-borne diseases in these and other countries with similar climatic characteristics.

## 1. Introduction

Climatic change strongly affects human and animal health increasing the risk of infections by many vector-borne parasitic, bacteria, and virus diseases [1, 2]. The temporal and spatial changes in temperature, rainfall, and humidity influence the distribution and seasonality of vectors and the extrinsic incubation of pathogens [3]. There is currently a considerable concern in the European institutions about the emergence or reemergence in the continent of some mosquito-borne diseases like malaria, leishmaniosis, dirofilariosis, West Nile virus, and chikungunga, among others, as a consequence of the climatic change [3, 4].

Dirofilariosis is a zoonotic disease mosaic transmitted by culicid mosquitoes, affecting dogs, cats, and humans, caused mainly by the filarid nematodes* D. immitis* and* D. repens* [5]. While animal dirofilariosis may result from a benign to a severe and potentially fatal disease [6], human dirofilariosis manifests itself as benign subcutaneous or pulmonary nodules that mimic malignant tumors [7] caused by immature worms. With increasing frequency in humans, fully developed* D. repens* adult worms are detected in conjunctival, retroocular, and intravitreal locations, responsible for loss of vision or other permanent ocular alterations [8, 9]. In addition, surgical removal of the nodules, very aggressive in pulmonary and intraocular/retroocular cases, can cause considerable damages and treatment costs [10, 11].

In Western Europe dirofilariosis has been historically considered endemic in the Mediterranean countries [12, 13], but during the last 14 years a rapid expansion into central and northern countries has occurred, mainly attributed to global warming [9]. In Eastern European countries dirofilariosis also exists but information on the epidemiological situation in canine populations is partial and limited. However, information is relatively abundant on human dirofilariosis, since many of the clinical cases recently reported in the world have been diagnosed in Ukraine and Russia, almost all attributed to* D. repens* [5, 14, 15]. Models to predict diseases patterns have become very valuable tools helping the design of appropriate control strategies [2, 16]. Unlike in other infectious diseases, in which predictions are difficult to assess [3, 17], changes predicted on dirofilariosis in Western Europe [18] were repeatedly confirmed, since its emergence in some central and northern European countries, previously free of dirofilariosis, has been already demonstrated [5]. Thus, dirofilariosis appears as a good model to evaluate the impact of global warming on the spread of mosquito-borne diseases. Furthermore, the influence of the changing climatic factors on the dynamic and trends of dirofilariosis has not been yet analyzed in the European far East, in spite of the strong thermal anomalies recently observed [19] and the recognition of dirofilariosis as an emerging disease [14, 15] in some post-Soviet states.

In the present work we developed a spatiotemporal geographic information system (GIS) model of* Dirofilaria* transmission in the former USSR based on the accumulated heat necessary to complete L3 development. Its validation was assessed by the spatial and temporal referentiation of human clinical cases obtained by an exhaustive retrospective review of different sources from 1981 to 2011 and derived morbidity. Moreover, a projection of the situation by 2030 is presented.

## 2. Materials and Methods

### 2.1. Data

We used daily temperature data recorded by a network of 421 meteorological stations of the Russian Federation and neighboring countries from 1981 to 2011 and temperatures foreseen by 2030 by the Russian Committee of Hydrometeorology [20].

Population data of each administrative unit of the former USSR were obtained from the current Russian State Federal Statistic Committee, State Statistics Committee of Ukraine, and the World Bank web site. Because the whole population appeared stable from 1981 to 2011 with a slight increase of 0.1%, we assumed the population data of 2011 for all calculations.

An exhaustive retrospective review of clinical cases of human dirofilariosis reported in the territory of the former USSR between 1981 and 2011 was carried out with the objective of the spatiotemporal referencing (year and administrative unit of occurrence) of each autochthonous case. The search was made using the following sources: (1) national and local medical journals from Russia and neighboring countries; (2) the archives of the Ukrainian Healthcare Ministry since 1997; (3) PubMed database to corroborate that no cases of human dirofilariosis have been published in non-Russian language journals was also consulted. Cases without date and/or geographical reference were excluded (8 cases). Clinical cases together with population data were used to calculate morbidity (number of cases/year/100000 people).

### 2.2. Model

The model predicts the spatiotemporal distribution of the number of generations of infective L3 of* Dirofilaria* that can yearly develop in the mosquito vectors (directly related to the annual length of the transmission period), considering temperatures calculated as indicated below. Full development of L3 needs 130 growing degree-days (GDDs) [16] accumulated in 30 consecutive days, the estimated mean life expectancy of a mosquito vector. Each day accumulates a number of GDDs resulting from the difference between the mean daily temperature and the threshold temperature for extrinsic incubation of* Dirofilaria* (L3 development), which has been already experimentally calculated in 14°C [21, 22]. Thus, the mean annual accumulated GDDs were used to calculate the number of annual generations of* Dirofilaria* developed in the vectors.

A GIS for the area studied was generated using the georeferenced meteostations previously indicated, the former Soviet Union regional administrative boundaries (oblasts and republics), and the model output databases. Given the high correlation between the indexes based on the GDD and altitude and latitude, the map of generations was produced following the methodology previously described [23–25]. In brief, considering the average GDDs previously calculated to each meteostation, for every 10-year period: 1981–1990, 1991–2000, 2001–2011, and on 2030, regressions were established to calculate the influence of altitude and latitude on GDD.

The GDD matrix was calculated using an image calculator module in accordance with the obtained regression formula, where corresponding latitudinal and altitudinal matrices were used as substitutes for latitude and altitude values. SRTM digital elevation models were used as altitudinal matrices [26]. The difference between calculated and real GDD values was the remainder. It was calculated as the difference between the regression calculated GDD and the real GDD for every meteorological station and interpolated over the whole analyzed territory by linear Kriging procedure. The interpolated remainder's layer was added to the calculated layer. The procedure was separately repeated for the coastal and continental stations. The coastal meteostations were located less than 30 km to the sea and the continental meteostations were located more than 30 km to the sea. The calculations were done separately because coastal and continental temperatures have different values and dynamic. After that, the coastal and continental GDD layers were joined through coastal 30 km buffer. The resulting GDD layers describing situation for 1981–1990, 1991–2000, 2001–2011, and 2030 have spatial resolution 10 km and presented in Albers Conic projection.

To validate the model two factors were analyzed: (1) the concordance between the predicted transmission areas and the spatiotemporal distribution of cases obtained by the retrospective review and (2) the statistical correlation between the number of predicted generations of* Dirofilaria* and the observed morbidity. This statistical correlation was evaluated globally and by decades using the Pearson correlation analysis, considering a significant correlation when *P* < 0.01 (highly significant).

Projection by 2030 scenario predicts the distribution of the transmission areas, including the number of predicted generations of* Dirofilaria,* and estimates the person-year of exposure using the same methodology described for the retrospective model.

## 3. Results

**Figure 1:**
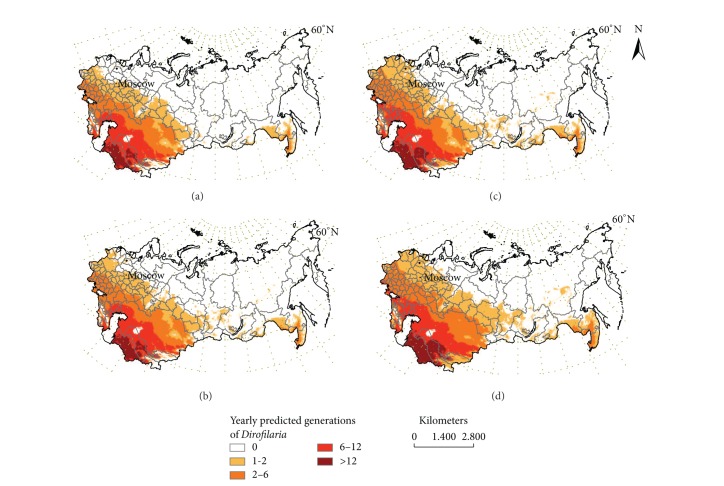
Retrospective prediction model of* Dirofilaria* transmission in the former USSR. Distribution of annual predicted generations: 1981–1990 (a), 1991–2000 (b), and 2001–2011 (c) and projection of the future scenario by 2030 (d).

The model generated retrospectively predicts the existence of two different potential transmission areas of dirofilariosis, one in the South West and other in the Asian Far East of the territory studied, where a different number of annual generations of* Dirofilaria* exist. Moreover, the model predicts significant changes over time (Figures 1(a), 1(b), and 1(c)). From 1981 to 1990, 1/3 of the territory studied becomes a suitable area for transmission. The Southwestern area covered from Ukraine to the Eastern borders of the Asian republics, excluding the Caucasus, Urals, and Tien Shan/Pamir ranges. In the northern part of this area, with the boundary in latitude 53–57°N in the European part of the Russian Federation and 61°N in Western Siberia, summer temperatures are low, optimal conditions for extrinsic incubation of* Dirofilaria* appear only sporadically, and 1 to 2 annual generations are predicted. In the Southern side high temperatures allow* Dirofilaria* extrinsic incubation during long periods each year, including two zones with 6–12 and more than 12 annual predicted generations, respectively. Between these zones there is another zone where annual predicted generations range from 2 to 6. In the Far East area 1 to 2 annual generations of* Dirofilaria* in most of the territory with a small zone with 2–6 annual generations are predicted. During the following two periods the boundary of the Southwestern area moved progressively until latitude 60°N in the European Northwestern side while it turned back slightly in Western Siberia. Both the zones with 2–6 and 6–12 predicted generations clearly extend to the Northwest. Scattered small areas with 1 to 2 predicted generations appear in Siberia as far as latitude 64°N (near the Polar Circle), along the Yenisei and Lena river basins, the Far East area remaining almost stable. Moreover, an altitudinal spreading also occurs, since some low lands of the Tien Shan/Pamir ranges become included in the predicted area. The increase of the area suitable for transmission (5.8% in 1991–2000 and 9% in 2001–2011) is accompanied by rises in the estimated population exposure of 7% and 7.4%, while person-year of exposure, respectively, rises 3.3% and 14.3% in the same periods (Table 1).

**Figure 2:**
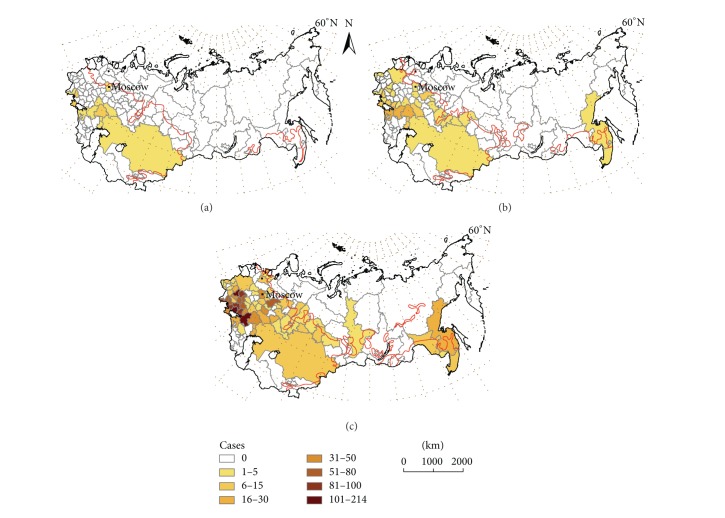
Administrative units of the former USSR where human dirofilariosis cases have been reported: 1981–1990 (a), 1991–2000 (b), and 2001–2011 (c). The red lines indicate the northern boundary of the predicted risk area of each lapse.

**Figure 3:**
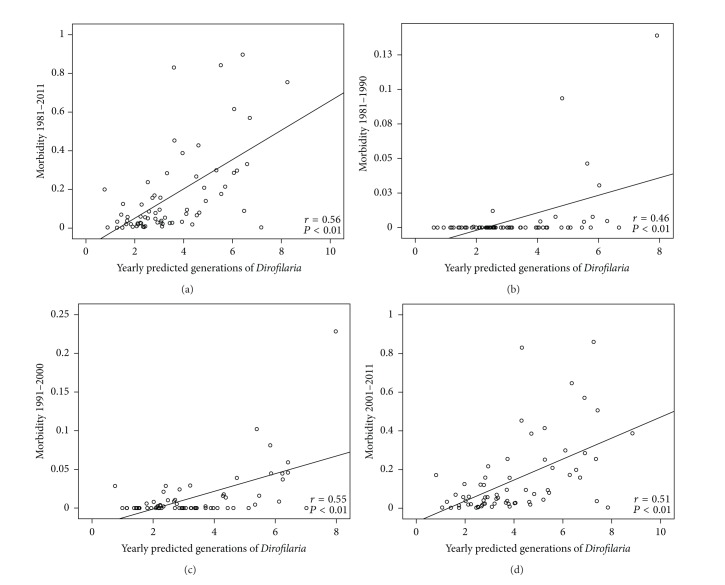
Statistical correlation (Pearson correlation coefficient) between yearly predicted generations of* Dirofilaria* and morbidity. From 1981 to 2011 (a), from 1981 to 1990 (b), from 1991 to 2000 (c), and from 2001 to 2011 (d).

**Figure 4:**
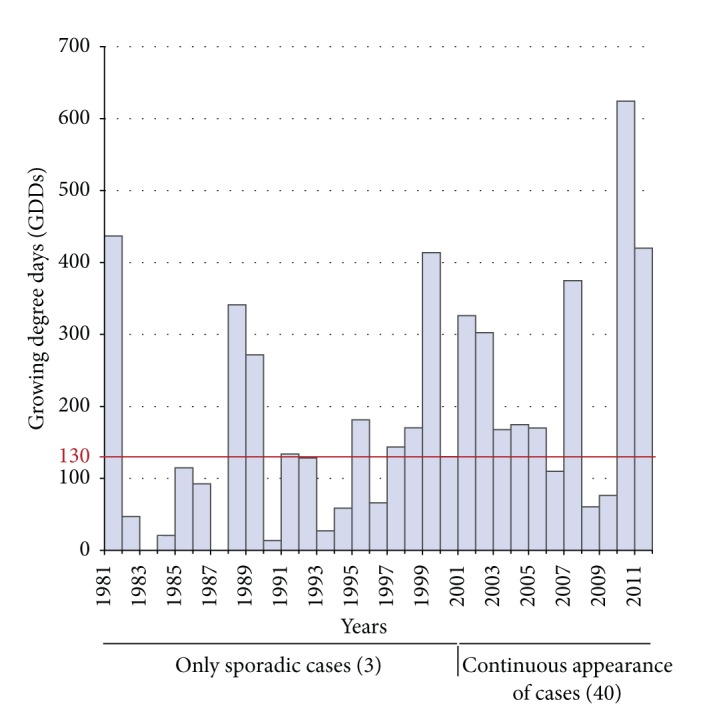
Appearance dynamics of human dirofilariosis cases and GDDs in Moscow administrative unit.

The retrospective review revealed 2154 cases of human dirofilariosis reported in the former USSR from 1981 to 2011. Data of geographical location, incidence, and morbidity are presented in Table 2. Fifty-eight, 196, and 1900 cases were, respectively, reported in 1981–1990, 1991–2000, and 2001–2011, most of them diagnosed in Ukraine and the Russian Federation. Cases appeared between latitudes 42°N (Kazakhstan) and 60°N (St. Petersburg) in 68 out of the 125 administrative units of the former USSR. Until 1996 there was a low annual incidence (2–11 cases/year), while from 1997 to 2011 incidence strongly rises from 22 to 365 cases/year. Morbidity rises over time in all administrative units where clinical cases have been referenced, except in Krasnodar (Southwestern Russia Federation), where reported cases decreased from 1991–2000 to 2001–2011 periods. Highest increases occurred in the Ukrainian and Russian administrative units near the Black and Azov seas, along the Dnieper, Don, and Volga river basins and in some regions of Southwestern Siberia. In relation to the validation of the model, the temporal and spatial distribution of cases reported in the literature review (by decades and administrative units) is shown in Figure 2. The concordance analysis between the predicted transmission areas and the spatiotemporal distribution of clinical cases shows that the model correctly predicts 97.10% of cases with a confidence interval (CI) of 92.42–100%. By periods the concordances are, respectively, 100% with CI of 96.15–100%, 96.97% with CI of 89.61–100%, and 98.53% with CI of 94.93–100%. The Pearson coefficient to assess the correlation between the number of predicted yearly generations and morbidity (Figure 3) shows a significant correlation at the global level (0.560, *P* < 0.01). By periods, correlations were 0.457 (1981–1990), 0.546 (1991–2000), and 0.510 (2001–2011), with *P* < 0.01 in all cases. The analysis revealed the existence of exceptional situations in very few administrative units, where higher or lower morbidities than expected appeared. Comparative dynamics of yearly appearance of the threshold of 130 GDDs allowing extrinsic incubation and incidence of cases was assessed in Moscow, as representative of the administrative units close to the North boundary of predicted area (Figure 4). From 1981 to 2000, when only 8 out of 20 years reached the threshold of 130 GDDs, 3 cases were recorded. From 2001 to 2011, with 8 out of 11 years reaching the threshold, 40 cases were reported in a yearly consecutive series. This series began after 4 consecutive years (1997–2000) reaching the threshold.

Projection by 2030 (Figure 1(d)) predicts extensive latitudinal and altitudinal changes in the potential transmission area (18.5% of surface area increase), with a 46% of the total territory and 110 out of 125 administrative units of the former USSR becoming included. These changes mainly occur in the northern zone with low number of predicted generations, the following being the most important: (1) the northward shift of the boundary exceeding latitude 60°N in all the Southwestern area; (2) the spreading of Siberian areas which have previously appeared; (3) the slight increase of the Far East area; (4) the continuing altitudinal spreading in low lands of the Urals and Tien Shan/Pamir ranges. Population exposure in 2030 will rise to 10.8%, affecting 92.4% of the total former USSR population, while the person-year of exposure will increase 11.1%, in respect of estimations of 2001–2011 period (Table 1).

## 4. Discussion

The effects of climatic change on the vector-borne diseases are greatest at the temperature range extremes at which transmission occurs (14°–18°C and 35°–40°C) [27]. Thus, important modifications in the distribution and seasonality of these diseases in temperate and cold areas where changes occur are expected [3]. The former USSR can be considered a paradigm of this fact because of its vast size and climatic variety, the strong thermal anomalies observed there in the last years [19, 28], the current recognition of human dirofilariosis as an emerging disease [14, 15], and precedents which have already demonstrated the emergence of different zoonotic diseases in the Russian Arctic [29].

In the present work a spatiotemporal model, based on climatic forecast using the concept of growing degree days [16], retrospectively predicts a latitudinal and altitudinal spreading of the potential transmission area of* Dirofilaria*, mainly in the Russian Federation and Ukraine, near the boundaries where temperatures are close to the threshold for extrinsic incubation of parasite larvae. Moreover, strong increases in the population exposure and person-year of exposure are also predicted, as a consequence of both the spreading trends into highly populated zones and the rise of the predicted yearly generations in Southwestern zones where dirofilariosis was already endemic. One of the most important problems related to prediction models is their correct validation, due to the frequent lack of suitable tools [17, 30]. To validate our model we used the clinical cases of human dirofilariosis identified by a retrospective review and derived morbidity in the studied territory from 1981 to 2011. In spite of clinical cases being only “the tip of the iceberg” of human dirofilariosis [31] their existence and dynamic of appearance can be good markers of the disease because, in endemic areas, human infections appear in parallel to canine dirofilariosis [32]. The retrospective review revealed that approximately 65% of the total cases of human dirofilariosis published in the world until 2011 were reported in the former USSR. Despite of this relatively abundant information, validation must be carefully analyzed. There exists a very high congruence between the transmission areas predicted by the model and the real spatiotemporal distribution of cases reported between 1981 and 2011 that confirms the spreading trend of the disease towards the Northwest of the area studied. Moreover, a significant statistical correlation between the number of annual predicted generations and morbidity exists. Nevertheless, there are some exceptions to the concordances observed, like the higher morbidity than expected in some Ukrainian and neighboring Russian administrative units and the lower morbidity than expected in some other Russian administrative units and Southern Asian republics. In the first case the situation can be attributed both to appropriate conditions for transmission and the awareness and wide experience of physicians in the differential diagnosis of the disease, which has led to numerous case reports. Moreover, human dirofilariosis is a notifiable disease from 1997 in Ukraine, a fact making these data very reliable. Otherwise, Asian republics have a very low hydrothermal coefficient (less than 1) [24] limiting the development of mosquito populations in most of their territory, which consist mainly of populated areas concentrated near the water bodies. Moreover, human dirofilariosis is not habitually included in the differential diagnosis of subcutaneous or pulmonary nodules in these countries. As a consequence, in spite of the high number of predicted generations of* Dirofilaria*, human cases are detected sporadically, but they will probably emerge when they become appropriately diagnosed, near the water bodies. Spreading of human dirofilariosis along the Dnieper, Don, and Volga river basins is consistent with the need of humidity as a key factor related to the abundance of mosquito populations. This fact also appears in the spreading predicted in Siberia where new risk areas emerge along the Yenisei and Lena river basins.

Emergence of human dirofilariosis in administrative units located closer to the northern boundary of the predicted area could be delayed in respect to the real introduction time, since consecutive appearance of cases seems to occur only after some years consecutively reaching the threshold allowing extrinsic incubation, as has been observed in Moscow. Otherwise morbidity rises have been detected between 2001 and 2011 in this and other administrative units near the northern boundary (see Table 2). Taken together, these two facts suggest that in spite of the apparent slow emergence of human dirofilariosis when suitable conditions for transmission occur sporadically or during short periods of time, once introduced and with suitable conditions, the disease can present a strong spreading potential.

Projection by 2030 maintains the geographical spreading pattern shown retrospectively by the model as well as the increase in the person-year of exposure, which occurs mainly in zones of the Southwestern area where 2–6 annual predicted generations are predicted. These predictions are consistent with the increase in temperatures foreseen by that time by the Russian Committee of Hydrometeorology. This agency predicts more moderate thermal increases than other organisms; thus, different scenarios could be taken in consideration. With these findings we can assume that human dirofilariosis morbidity will increase in the future, emerging in many northern cold administrative units of the European Russia, in low lands of the ranges and in Siberia as far as latitude 64°N. Moreover, morbidity will strongly increase in Southwestern zones with medium number of predicted yearly generations.

## 5. Conclusion

Our model suggests that regional warming is clearly associated with the long-term spreading and emergence of dirofilariosis in extensive territories of the former USSR confirmed by the spatiotemporal appearance pattern of cases from 1981 to 2011. Nevertheless, factors influencing the dynamic of vector-borne diseases are complex, so prediction models necessarily must simplify the real situation. Our analysis suggests the involvement of other factors not included in the model like humidity, population distribution, knowledge of and interest in the disease by the scientific community, and probably pets management (there is a huge stray dogs population lacking preventive measures in the former USSR acting as reservoirs), among others. Our findings, together with the demonstrated severity of many cases, suggest that human dirofilariosis has become a serious medical problem that will increase in the future; thus, control of reservoir infections is urgently needed. Our model can help to design appropriate preventive and control strategies in the post-Soviet states. Moreover, these results can alert about the epidemiological behavior of this and other mosquito-borne diseases, not analyzed until now in different areas of the world.

## Figures and Tables

**Table 1:** Estimated area, population exposure, and transmission of *Dirofilaria* in the former USSR.

Annual predicted generations of *Dirofilaria* intervals	1981–1990			1991–2000			2001–2011			2030		
	Area (×1000 km^2^)	Population exposure (millions)	PYE (millions)	Area (×1000 km^2^)	Population exposure (millions)	PYE (millions)	Area (×1000 km^2^)	Population exposure (millions)	PYE (millions)	Area (×1000 km^2^)	Population exposure(millions)	PYE (millions)
0	14908	77.15	0	14467 (−3%)	62.83 (−18.6%)	0 (0%)	13740 (−5%)	46.54 (−25.9%)	0 (0%)	12115 (−11.8%)	21.49 (−53.8%)	0 (0%)
0–2	2936	92.45	138.68	3321 (13.1%)	103.69 (12.2%)	155.54 (12.2%)	3641 (9.6%)	100.70 (−2.9%)	151.05 (−2.9%)	4388 (20.5%)	103.13 (2.4%)	154.70 (2.4%)
2–6	2498	62.19	248.76	2534 (1.4%)	65.27 (5%)	261.08 (5%)	2619 (1.6%)	76.69 (17.5%)	306.76 (17.5%)	3153 (20.4%)	96.27 (25.5%)	385.08 (25.5%)
6–12	1735	38.98	350.82	1733 (−0.1%)	38.98 (0%)	350.82 (0%)	1918 (10.7%)	40.79 (40.6%)	367.11 (40.6%)	2068 (7.8%)	41.68 (2.2%)	375.12 (2.2%)
>12	455	11.87	142.44	477 (4.8%)	11.87 (0%)	142.44 (0%)	614 (28.72%)	17.91 (50.9%)	214.92 (50.9%)	808 (31.6%)	20.05 (11.9%)	240.6 (11.9%)
Total affected area	7624	205.48	880.70	8065 (5.8%)	219.80 (7%)	909.88 (3.3%)	8792 (9%)	236.09 (7.4%)	1039.84 (14.3%)	10417 (18.5%)	261.49 (10.8%)	1155.5 (11.1%)

PYE: person-year of exposure. % increase shown in parentheses.

**(a):** 

	Population	1981–1990		1991–2000		2001–2011	
		Cases	Morbidity	Cases	Morbidity	Cases	Morbidity
Russia							
St-Petersburg	4600276	0	0.000	0	0.000	1	0.002
Krasnoyarsk	2893926	0	0.000	0	0.000	1	0.003
Perm	2701174	0	0.000	0	0.000	9	0.033
Kirov	1391059	0	0.000	0	0.000	8	0.058
Sverdlovsk	4393797	0	0.000	0	0.000	1	0.002
Novgorod	640613	0	0.000	0	0.000	8	0.125
Yaroslavl	1306320	0	0.000	0	0.000	5	0.038
Tyumen	3430313	0	0.000	2	0.006	5	0.015
Ivanovo	1066541	0	0.000	0	0.000	13	0.122
Mari-El	698176	0	0.000	2	0.029	4	0.057
Nizhni-Novgorod	3323600	0	0.000	7	0.021	72	0.217
Omsk	2012092	0	0.000	0	0.000	12	0.060
Vladimir	1430084	0	0.000	0	0.000	8	0.056
Moscow	17315765	1	0.001	2	0.001	40	0.023
Kurgan	947566	0	0.000	1	0.011	15	0.158
Tatarstan	3778504	0	0.000	2	0.005	9	0.024
Novosibirsk	2649871	0	0.000	0	0.000	5	0.019
Smolensk	965986	0	0.000	0	0.000	2	0.021
Khabarovsk	1400425	0	0.000	4	0.029	24	0.171
Chelyabinsk	3508447	0	0.000	1	0.003	1	0.003
Bashkortostan	4065993	0	0.000	1	0.002	2	0.005
Kaluga	1001559	0	0.000	0	0.000	1	0.010
Mordovia	826526	0	0.000	0	0.000	4	0.048
Ryazan	1151439	0	0.000	1	0.009	8	0.069
Amur	860686	0	0.000	0	0.000	6	0.070
Samara	3170141	0	0.000	0	0.000	30	0.095
Penza	1373236	0	0.000	4	0.029	35	0.255
Altay	2490714	3	0.012	6	0.024	30	0.120
Tambov	1088437	0	0.000	0	0.000	3	0.028
Lipetsk	1157852	0	0.000	0	0.000	3	0.026
Orenburg	2112910	0	0.000	0	0.000	4	0.019
Saratov	2564835	2	0.008	10	0.039	24	0.094
Kursk	1148610	0	0.000	0	0.000	1	0.009
Voronezh	2261628	1	0.004	4	0.018	10	0.044
Belgorod	1530124	0	0.000	0	0.000	5	0.033
Volgograd	2589887	12	0.046	21	0.081	41	0.158
Biribidzhan	185039	0	0.000	0	0.000	1	0.054
Rostov	4229505	2	0.005	27	0.064	214	0.506
Astrakhan	1007113	14	0.139	23	0.228	39	0.387
Krasnodar	5160656	4	0.008	23	0.045	19	0.037
Vladivostok	1981970	0	0.000	2	0.010	8	0.040
Stavropol	2711198	0	0.000	0	0.000	1	0.004
Ukraine							
Chernihiv	1083827	0	0.000	0	0.000	90	0.830
Sumy	1147749	0	0.000	0	0.000	52	0.453
Rivne	1156009	0	0.000	0	0.000	11	0.095
Volyn	1040606	0	0.000	0	0.000	3	0.029
Zhytomyr	1270939	0	0.000	0	0.000	20	0.157
Kiev	4536061	0	0.000	1	0.002	175	0.386
Lviv	2539031	0	0.000	1	0.004	6	0.024
Kharkiv	2732086	0	0.000	0	0.000	57	0.209
Khmelnytskyi	1318377	0	0.000	0	0.000	5	0.038
Poltava	1472541	0	0.000	2	0.014	61	0.414
Cherkasy	1274125	0	0.000	2	0.016	32	0.251
Crimea	1963770	6	0.031	9	0.046	50	0.255
Luhansk	2263676	0	0.000	1	0.004	39	0.172
Vinnytsia	1631305	0	0.000	0	0.000	12	0.074
Ivano-Frankivsk	1381184	0	0.000	0	0.000	1	0.007
Kirovohrad	999285	0	0.000	0	0.000	8	0.080
Zakarpattia	1252608	0	0.000	1	0.008	2	0.016
Dnipropetrovsk	3312064	0	0.000	0	0.000	99	0.299
Chernivtsi	905189	0	0.000	0	0.000	2	0.022
Donetsk	4387702	0	0.000	7	0.016	87	0.198
Zaporizhia	1786905	0	0.000	8	0.045	102	0.571
Mykolaiv	1175598	11	0.094	12	0.102	76	0.646
Odessa	2387282	1	0.004	2	0.008	68	0.285
Kherson	1081336	0	0.000	4	0.037	93	0.860

**(b):** 

Post-Soviet	Population	1981–1990		1991–2000		2001–2011	
states		Cases	Morbidity	Cases	Morbidity	Cases	Morbidity
Belarus	9457500	0	0.000	2	0.002	7	0.007
Kazakhstan	16004800	1	0.001	1	0.001	10	0.006

**(c):** 

Total cases	1981–1990	1991–2000	2001–2011	Total
Russia	39	143	732	914
Ukraine	18	50	1151	1219
Post-Soviet states	1	3	17	21
USSR	58	196	1900	2154

## References

[1] Core Writing Team, Pachauri RK, Reisinger A, IPCC (2007). Climate change 2007: synthesis report. *Contribution of Working Groups I, II and III to the Fourth Assessment Report of the Intergovernmental Panel on Climate Change*.

[2] McMichael AJ, Woodruff RE, Hales S (2006). Climate change and human health: present and future risks. *The Lancet*.

[3] Semenza JC, Menne B (2009). Climate change and infectious diseases in Europe. *The Lancet Infectious Diseases*.

[4] EASAC Climate change and infectious diseases in Europe. http://www.easac.eu/home/reports-and-statements/detail-view/article/climate-chan.html.

[5] Simón F, Siles-Lucas M, Morchón R (2012). Human and animal dirofilariasis: the emergence of a zoonotic mosaic. *Clinical Microbiology Reviews*.

[6] McCall JW, Genchi C, Kramer LH, Guerrero J, Venco L (2008). Chapter 4 heartworm disease in animals and humans. *Advances in Parasitology*.

[7] Simón F, Morchón R, González-Miguel J, Marcos-Atxutegi C, Siles-Lucas M (2009). What is new about animal and human dirofilariosis?. *Trends in Parasitology*.

[8] Avdiukhina TI, Lysenko AI, Supriaga VG, Postnova VF (1996). Dirofilariasis of the vision organ: registry and analysis of 50 cases in the Russian Federation and in countries of the United Independent States. *Vestnik oftalmologii*.

[9] Genchi C, Kramer LH, Rivasi F (2011). Dirofilarial infections in Europe. *Vector-Borne and Zoonotic Diseases*.

[10] Ro JY, Tsakalakis PJ, White VA (1989). Pulmonary dirofilariasis: the great imitator of primary or metastatic lung tumor. A clinicopathologic analysis of seven cases and a review of the literature. *Human Pathology*.

[11] Ilyasov B, Kartashev V, Bastrikov N, Morchón R, González-Miguel J, Simón F (2013). Delayed diagnosis of dirofilariasis and complex ocular surgery, Russia. *Emerging Infectious Diseases*.

[12] Genchi C, Kramer LH, Prieto G, Simón F, Genchi C (2001). Epidemiology of canine and feline dirofilariasis: a global view. *Heartworm Infection in Humans and Animals*.

[13] Pampiglione S, Rivasi F, Genchi C, Rinaldi L, Cringoli G (2007). Human dirofilariasis to Dirofilaria (Nochtiella) repens: an update of world literature from 1995–2000. *Dirofilaria Immitis and D. Repens in Dog and Cat and Human Infections*.

[14] Kramer LH, Kartashev VV, Grandi G (2007). Human subcutaneous dirofilariasis, Russia. *Emerging Infectious Diseases*.

[15] Kartashev V, Batashova I, Kartashov S (2011). Canine and human dirofilariosis in the Rostov Region (Southern Russia). *Veterinary Medicine International*.

[16] Rinaldi L, Musella V, Biggeri A, Cringoli G (2006). New insights into the application of geographical information systems and remote sensing in veterinary parasitology. *Geospatial Health*.

[17] Tanser FC, Sharp B, Le Sueur D (2003). Potential effect of climate change on malaria transmission in Africa. *The Lancet*.

[18] Genchi C, Rinaldi L, Mortarino M, Genchi M, Cringoli G (2009). Climate and *Dirofilaria* infection in Europe. *Veterinary Parasitology*.

[19] http://www.noaa.gov/.

[20] Federal Service for Hydrometeorology and Environmental Monitoring (2008). *Evaluation Report on Climate Change and Its Impact on the Russian Federation*.

[21] Fortin JF, Slocombe JOD (1981). Temperature requirements for the development of *Dirofilaria immitis* in *Aedes triseriatus* and *Ae. vexans*. *Mosquito News*.

[22] Lok JB, Knight DH, Seward RL, Knight DH Laboratory verification of a seasonal heartworm transmission model.

[23] Greene SL, Hart TC, Afonin A (1999). Using geographic information to acquire wild crop germplasm for ex situ collections: I. Map development and field use. *Crop Science*.

[24] Afonin AN, Greene SL, Dzyubenko NI, Frolov AN http://www.agroatlas.ru/.

[25] Simón L, Afonin A, López-Díez LI (2014). Geo-environmental model for the prediction of potential transmission risk of *Dirofilaria* in an area with dry climate and extensive irrigated crops. The case of Spain. *Veterinary Parasitology*.

[26] Jarvis A, Reuter HI, Nelson A, Guevara E http://srtm.csi.cgiar.org/.

[27] Githeko AK, Lindsay SW, Confalonieri UE, Patz JA (2000). Climate change and vector-borne diseases: a regional analysis. *Bulletin of the World Health Organization*.

[28] Berkeley Earth http://Berkeleyearth.org/.

[29] Revich B, Tokarevich N, Parkinson AJ (2012). Climate change and zoonotic infections in the Russian Arctic. *International Journal of Circumpolar Health*.

[30] Semenza JC, Suk JE, Estevez V, Ebi KL, Lindgren E (2012). Mapping climate change vulnerabilities to infectious diseases in Europe. *Environmental Health Perspectives*.

[31] Simón F, López-Belmonte J, Marcos-Atxutegi C, Morchón R, Martín-Pacho JR (2005). What is happening outside North America regarding human dirofilariasis?. *Veterinary Parasitology*.

[32] Montoya-Alonso JA, Mellado I, Carretón E, Cabrera-Pedrero ED, Morchón R, Simón F (2010). Canine dirofilariosis caused by *Dirofilaria immitis* is a risk factor for the human population on the island of Gran Canaria, Canary Islands, Spain. *Parasitology Research*.

